# Mangrove plant, *Rhizophora mucronata* (Lamk, 1804) mediated one pot green synthesis of silver nanoparticles and its antibacterial activity against aquatic pathogens

**DOI:** 10.1186/2046-9063-8-11

**Published:** 2012-05-18

**Authors:** Jaganathan Umashankari, Dhinakarasamy Inbakandan, Thipramalai T Ajithkumar, Thangavel Balasubramanian

**Affiliations:** 1 CAS in Marine Biology, Annamalai University, Parangipettai, Tamilnadu, 608502, India; 2 Center for Ocean Research, Sathyabama University, Chennai, Tamilnadu, 600 119, India

**Keywords:** Silver nanoparticles, *Rhizophora mucronata*, One pot green synthesis, Antimicrobial, Aquatic pathogens

## Abstract

**Background:**

Biosynthesis of nanoparticles has received increasing attention due to the growing need to develop safe, time-effective and environmentally friendly technologies for nano-materials synthesis. This paper reports the one pot green synthesis of silver nanoparticles (AgNPs) using the leaf bud extract of a mangrove plant, *Rhizophora mucronata* and their antimicrobial effects against aquatic pathogens. Highly stable AgNPs were synthesized by treating the mangrove leaf bud extract with aqueous silver nitrate solution at 15 psi pressure and 121°C for 5 minutes.

**Results:**

The biosynthesized AgNPs were characterized by UV-visible spectrum, at 426 nm. The X-Ray Diffraction (XRD) pattern revealed the face-centered cubic geometry of AgNPs. Fourier Transform Infra Red (FTIR) spectroscopic analysis was carried out to identify the possible biomolecules responsible for biosynthesis of AgNPs from the leaf bud extract. The size and shape of the well-dispersed AgNPs were documented with the help of High Resolution Transmission Electron Microscopy (HRTEM) with a diameter ranged from 4 to 26 nm. However a maximum number of particles were observed at 4 nm in size. The antibacterial effects of AgNPs were studied against aquatic pathogens *Proteus spp.*, *Pseudomonas fluorescens* and *Flavobacterium spp*., isolated from infected marine ornamental fish, *Dascyllus trimaculatus*.

**Conclusion:**

This study reveals that the biosynthesized AgNPs using the leaf bud extract of a mangrove plant (*R. mucronata)* were found equally potent to synthetic antibiotics. The size of the inhibition zone increases when the concentration of the AgNPs increased and varies according to species.

## Background

In recent years the green processes for the synthesis of silver nanoparticles (AgNPs) is evolving into an important branch of nanotechnology and it subsist to be a valuable science [1,2]. The AgNPs are applicable in purifying drinking water, degrading pesticides and killing human pathogenic bacteria [3] etc. Nanoparticles have become more significant in recent years and have created much impact in the areas of chemical, electronic, and biological sciences. Although such particles can be synthesized by physical, chemical and biological methods in the past few years, among them biological method has gained more importance [4,5]. Recently the use of plant extracts act as an effective agent against various disease causing microorganisms including plant pathogens. The organic and inorganic nanosized particles are finding increasing attention in medical applications [6] due to their amenability to biological functionalization. Based on enhanced effectiveness, the new age drugs are nanoparticles of polymers, metals or ceramics, which can fight against conditions like cancer [7] and kill human pathogens like bacteria [8-10]. At the present time, plant-mediated biological synthesis of nanoparticles is gaining more importance due to its simple experimental procedure and eco-friendliness [11].

The antimicrobial potential of AgNPs is also applicable in immense area of biology and medicine, in which the physiochemical property and strong toxicity of AgNPs to microorganisms is more pertained. The antimicrobial activity of AgNPs against, *Escherichia coli* as a model of Gram negative bacteria was also illustrated [8]. Similarly biosynthesis of AgNPs using bacteria [12-14], fungi [15-17], yeast [18] and plants [19-21] were also well renowned. Recently, AgNPs have been synthesized using various plants like, *Acalypha* indica [22], *Pelargonium graveolens*[23], *Parthenium hysterophorus*[24], *Aloe barbadensis*[25] and *Gliricidia sepium*[26] Hypocotyls, collar and bark of *Rhizophora mucronata* and other species of mangrove plants have shown an enhanced antimicrobial activity against human urinary tract infections caused by bacterial pathogens [27]. However, the leaf buds of *R. mucronata* extract have no antibacterial activity, but it plays a major role after the biosynthesis of AgNPs. Hence, the present study aims to investigate the one pot green synthesis potential of AgNPs from extract of *R. mucronata* and their antimicrobial effect on selected aquatic pathogens.

## Methods

### Materials

All analytical chemicals such silver nitrate (AgNO_3_) was purchased from Merck Chemicals, India and media components were purchased from Hi-Media, Mumbai, India. All the aqueous solutions were prepared using triple distilled de-ionized water. Fresh leaf buds of *R. mucronata* were collected from the mangrove which is situated in the vicinity of Vellar estuary, Porto Novo, (Lat.11° 29’N; Long 79° 46’E) in the south east coast of India.

### One pot green synthesis

Leaf buds weighing 15 g were thoroughly washed using sterile distilled water and ground well using mortar and pestle. The well grounded material was mixed with 100 mL of sterile distilled water and then transferred in 500 mL Erlenmeyer flask and the content was boiled for 3 minutes. After boiling the content was filtered using cheese cloth filter and the filtrate was stored in a sterile beaker at 4°C for further use. 100 mL of aqueous silver nitrate (1 mM) was added with 10 mL of the leaf extract of *R. mucronata*. This mixture was kept in 15 psi pressure at 121°C for 5 minutes. A color change from green to yellowish brown, visually confirms the formation of AgNPs. This one pot green synthesis was the modified method followed by Vigneshwaran et al. 2006 [28].

### Nanoparticle characterization

The resulting solution was then diluted with a small aliquot of 100 μL of the sample with 1 mL de-ionized water and assayed in UV Visible spectroscopy. UV Visible spectral analysis has been done to know the surface plasmon resonances band by using Perkin Elmer UV-visible absorption spectrophotometer with the resolution of 1 nm between 200 and 800 nm, possessing a scanning speed of 300 nm/minutes. The reduction of pure Ag^+^ ions to form AgNPs using mangrove extract was characterized by UV-Visible spectrum of the reaction medium.

X ray diffraction (XRD) pattern analysis was done to know the face center cubic crystalline nature of the nanoparticles. The biosynthesized AgNPs using mangrove extract was lyophilized to powder. The powdered or dried AgNPs were coated on XRD grid and the spectra was recorded by using by Rich seifert p 300 instrument operated at a voltage of 40 KV and a current of 30 mA with Cu Kα radiation.

The Fourier transform infrared spectroscopy measurements were carried out to identify the possible biomolecules for the biosynthesis of AgNPs. Dry powder of the biomass and AgNPs solution were centrifuged at 5000 rpm for 30 min and resulting pellet was re-suspended in sterile distilled water. The dispersed solution was lyophilized to make it as fine particles. These particles are used to make KBr pellets. The measurements were carried out from 4000 to 400 cm^-1^ using Perkin Elmer infrared spectroscopy.

High Resolution Transmission Electron Microscopy (HR-TEM) was used to analyze the AgNPs which were recorded by placing a drop of the suspension on carbon-coated copper grids and allowing the water to evaporate. Samples were prepared by drop coating AgNPs solutions onto carbon coated copper TEM grids. The films on the TEM grids were allowed to stand for 2 minutes following which the extra solution was removed using a blotting paper and the grid was allowed to dry, prior to the measurement. The observations of TEM were performed on JEOL 3010 operated at an accelerating voltage of 120 KV.

### Antimicrobial activity

The one pot green synthesized AgNPs were tested for antimicrobial effect against marine aquatic pathogens, *Proteus spp.Pseudomonas florescence* and *Flavobacterium spp.*, The fish pathogens were obtained from the Marine ornamental fish hatchery, Annamalai University. These pathogens were isolated from infected marine ornamental fish, *Dascyllus trimaculatus* and reported by Dhayanithi et al. 2010 [29]. Wells were made on the agar plates using a gel puncture to about 10 mm diameter in Muller Hinton agar medium. Strains were swabbed uniformly onto the individual plates using sterile cotton swabs. 100 μg of lyophilized AgNPs were dispersed in 100 μL of distilled water. The dispersed solution was impregnated in the well at 25, 50 and 75 μL, to get the concentration of 25, 50 and 75 μg/μL respectively, to perform the well diffusion method. Chloramphenicol (1 ppm) is used as a positive control and plain leaf bud extract (*R. mucronata*) as a negative control. After 24 h, the diameters of inhibition zones around the wells were measured in millimeter. The size of the circular inhibition zone is directly proportional to the antimicrobial effect of the biosynthesized AgNPs against marine microbial pathogens [30,31].

## Results and discussion

**Figure 1:**
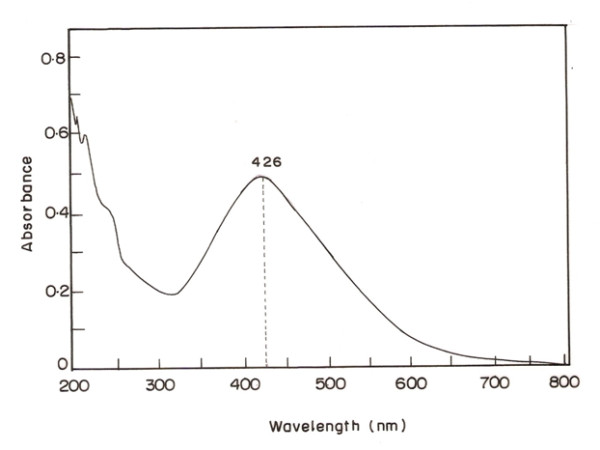
UV-Visible spectra of Silver nanoparticles synthesized from aqueous leaf bud extract of R.mucronata.

In this present study we focused on the one pot green synthesis of AgNPs using a leaf bud extract of *R. mucronata* which is simple, convenient and eco-friendly. The green synthesis of AgNPs has been investigated as an alternative to chemical and physical ones. A conical flask containing the extract of mangrove leaf bud *R. mucronata* and aqueous silver nitrate (1 mM) was kept at 121°C for 5 minutes and the resulting solution was turned to yellowish brown, this color change was due to excitation of surface plasmon vibrations in the metal nanoparticles. This visible observation indicates the reduction of the Ag^+^ ions and the biosynthesis of AgNPs. This observation was further reconfirmed by UV-visible spectrum and XRD analysis. The (Figure 1) shows the UV visible spectra recorded from the reaction medium which were characterized by UV–vis spectrophotometer; it is well observed that the silver surface plasmon resonance band occurs at 426 nm.

In metal nanoparticles such as in silver, the conduction band and valence band lie very close to each other and through these electrons move freely. These free electrons give rise to a surface plasmon resonance (SPR) absorption band, occurring due to the collective oscillation of electrons of AgNPs in resonance with the light wave. Classically, the electric field of an incoming wave induces polarization of the electrons with respect to much heavier ionic core of AgNPs. As a result a net charge difference occurs, which in turn acts as a restoring force. This creates a dipolar oscillation of all the electrons with the same phase. When the frequency of the electromagnetic field becomes resonant with the coherent electron motion, a strong absorption takes place, which is the origin of the observed color, which was yellowish brown in our observation. This absorption strongly depends on the particle size, dielectric medium and chemical surroundings. The UV/Vis absorption spectra of the silver nano particles dispersed in *R. mucronata* extracts is shown in Figure 1. The absorption peak (SPR) was obtained in the visible range at 426 nm, the broad spectra is due to the size (4 nm) and shape (spherical) of the biosynthesized AgNPs which were documented by High Resolution TEM micrograph.

**Figure 2:**
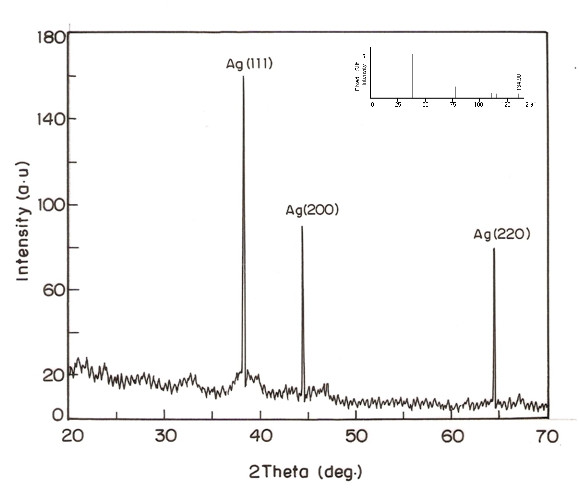
**XRD patterns of the silver nanoparticles synthesized from aqueous leaf extract of R. mucronata.** (Inset showing the standard XRD pattern for silver: JCPDS 652871)

The result of XRD patterns (Figure 2) showed AgNPs are reflected in the 2θ on 38.1, 44.3 and 64.4 that respect the Bragg model of diffraction. The peak corresponding to the 2θ = 38.1°(111), 44.3°(200) and 64.4°(220) of the sample respects the JCPDS 652871 and it was confirmed the crystalline nature of the AgNPs, obtained biogenic AgNPs which indexed the planes 111, 200 and 220 of the face center cubic nature of silver nanopartilces. The angle measured by this method corresponds to the lattice planes were observed the face center cubic structures of silver matched with the database of Joint Committee on Powder Diffraction Standard [32]. Thus XRD patterns clearly showed that the AgNPs formed by the reduction of Ag^+^ ions by the extract of *R. mucronata* with aqueous silver nitrate are crystalline in nature.

**Figure 3:**
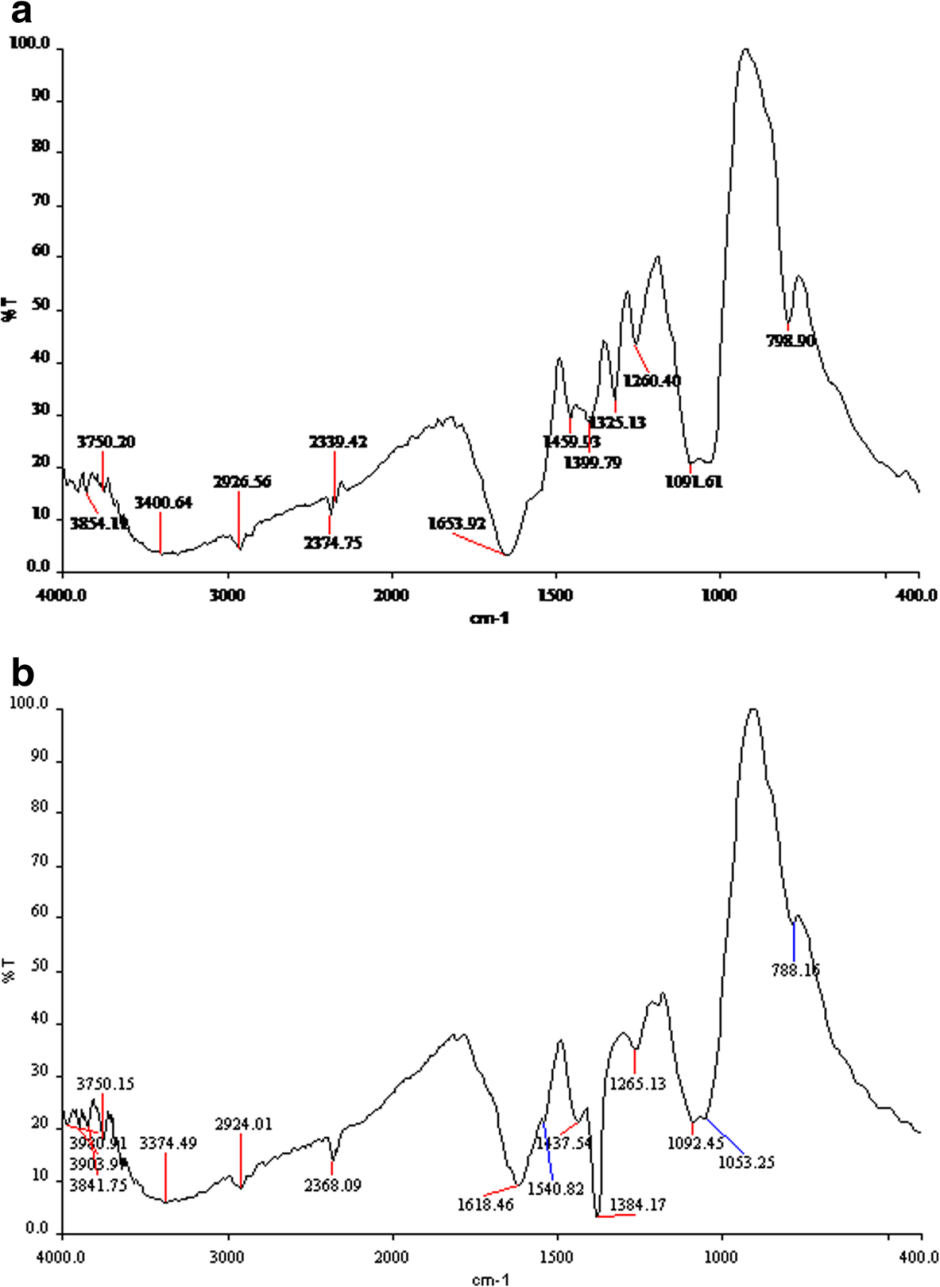
**(a) FT-IR spectra of plain R. mucronata. (b)** Biologically synthesized silver nanoparticles using R. mucronata.

FTIR measurements were carried out to identify the possible biomolecule responsible for the reduction of the Ag^+^ ions and capping of the bioreduced AgNPs synthesized by mangrove leaf bud extract *R. mucronata.* The FTIR spectrum shows peaks at 2368, 1618, 1540, 1384, 1325, 1265, 1053 and 788 cm^-1^. Curve of the mangrove leaf bud extract of *R. mucronata* (Figure 3a) resulted a multiple broad peaks at 2368 cm^-1^ corresponding to N-H stretching of any ammonium ions; the medium band at 1618 cm^-1^ corresponding to stretching of C = N; the stronger band at 1540 cm^-1^ corresponding to N-O stretching of nitro compounds. The weaker band at 1384 cm^-1^ corresponding to N-O stretching of nitro compounds; the broad band at 1092 cm^-1^ corresponding to C-X stretching of fluoroalkanes; the strong band at 788 cm^-1^ corresponding to C-H stretching of aromatic benzene.1325 cm^-1^ relating the stretching of amide.

Curve of AgNPs biosynthesized using the mangrove leaf bud extract of *R.mucronata* (Figure 3b) resulted a broad band at 3396 cm^-1^ corresponding to O-H stretching of high concentration of alcohols or phenols; the multiple broad band at 2375 cm^-1^ corresponding to N-H stretching of ammonium ions; the medium band at 1636 cm^-1^ corresponding to stretching of C = N; the weak to strong band at 1444 cm^-1^ corresponding to C-C stretching of aromatic C = C. the medium band at 1264 cm^-1^ corresponding to C-O stretching of any carboxylic acids. A band shift from 1094 cm^-1^ corresponding to C-X stretching of ordinary fluroalkanes to strong band at 798 cm^-1^ corresponding to C-H stretching of benzene of aromatic compounds. The disappearance of 1325 cm^-1^ relating the stretching of amide which cause the capping of AgNPs.

FTIR spectroscopy from the absorption of IR radiation through resonance of non-centro symmetric (IR active) modes of vibration and is a useful tool for quantifying secondary structure in metal nanoparticle–biomolecules interaction. Figure 3a-b confirmed that the N-H stretching vibration of primary amines and C-N stretching and over lapping of aliphatic amines has the stronger ability to bind metal, so that the secondary metabolites from mangrove leaf bud extract of R.mucronata could most possibly form a coat covering the metal nanoparticles (i.e. capping of silver nanoparticles) to prevent agglomeration of the particles and stabilizing in the medium. This evidence suggests that the biological molecules could possibly perform the function for the formation and stabilization of the silver colloids in aqueous medium. The exact mechanism leading to the reduction of metal ions is yet to be elucidated for mangrove leaf bud extract of R.mucronata.

**Figure 4:**
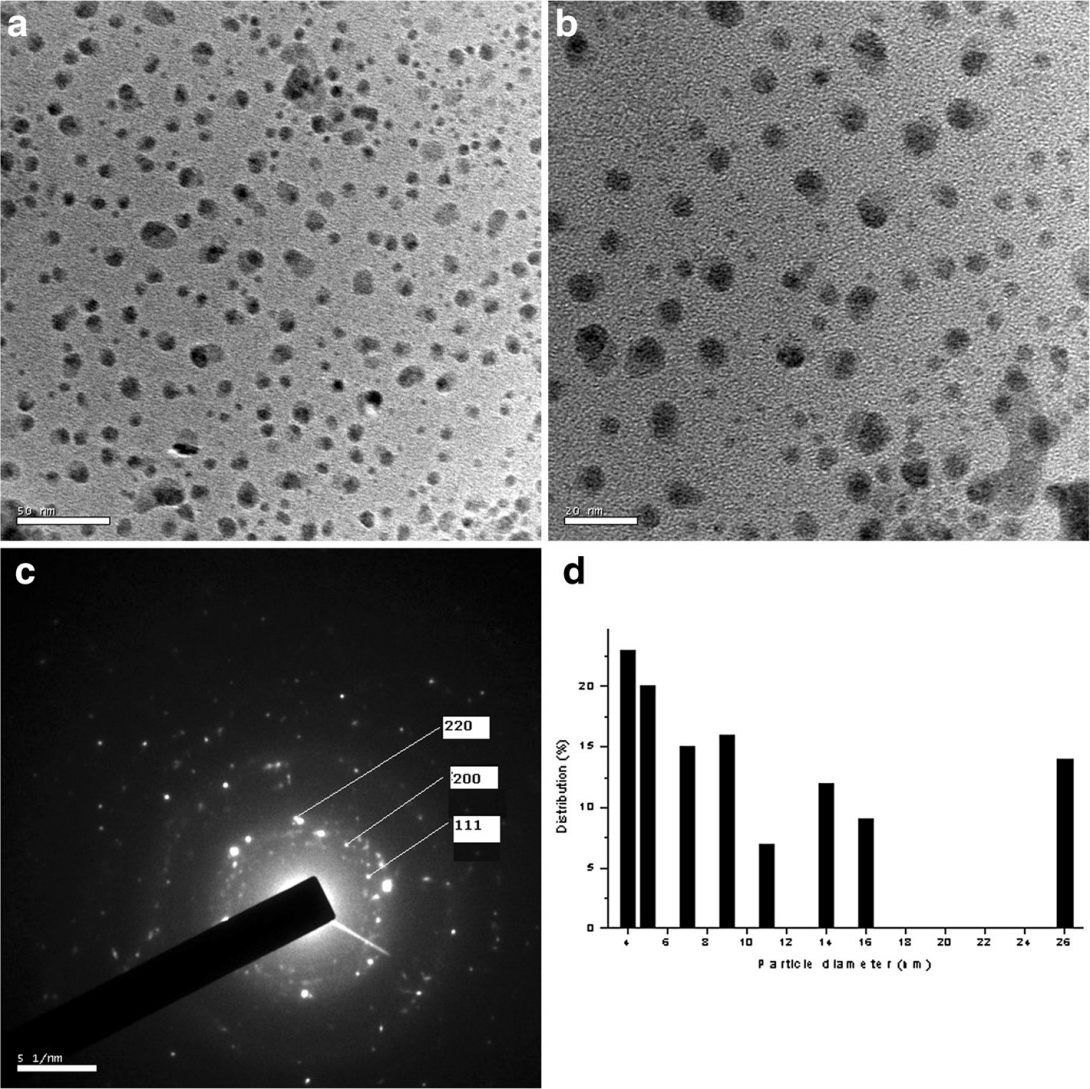
**TEM images of silver nanoparticle in two magnifications.(a)** 50 nm **(b)** 20 nm **(c)** SAED pattern and **(d)** Histogram showing the particle size distribution.

The size and shape of the biosynthesized AgNPs were documented by High Resolution TEM micrograph (Figure 4a, b, and c). The enlarged TEM graphs helped to plot a histogram (Figure 4d), which revealed the particle distribution according to the size. The TEM micrograph of the AgNPs confirmed that the particles were likely to be spherical in shapes with a size range from 4 to 26 nm. However a maximum number of particles were observed at 4 nm in size. The selected area electron diffraction (SAED) pattern of the nanoparticles explained the face centered cubic (fcc) crystalline structure of silver with different diffracting index (Figure 4c).

**Figure 5:**
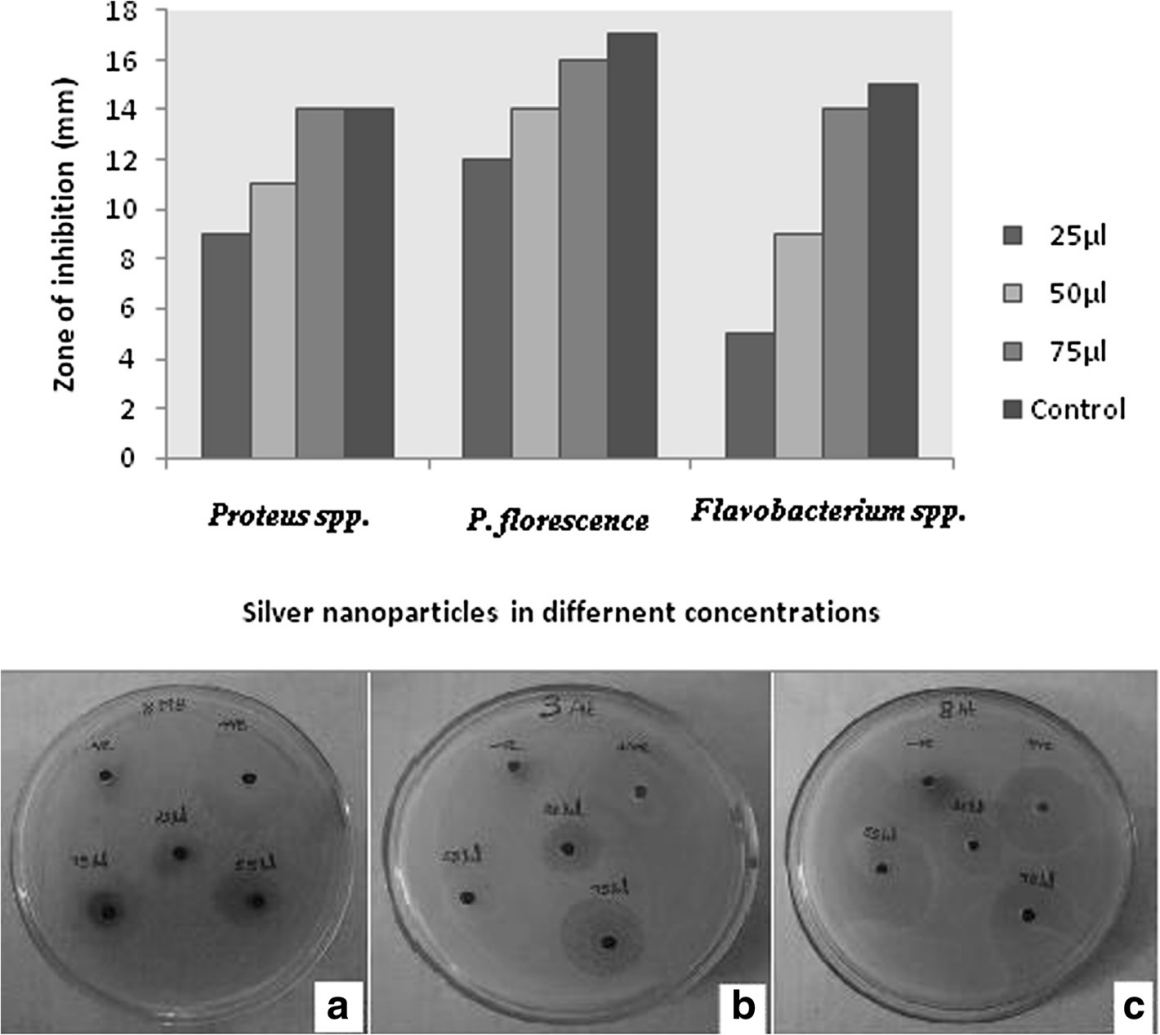
**Graph showing the rate of inhibition of silver nanoparticles against marine ornamental fish pathogens.** (Inset showing the antimicrobial activity against **(A)*** Proteus spp.*, **(B)*** Pseudomonas florescence* and **(C)*** Flavobacterium spp.*

Antimicrobial effects of one pot green synthesized AgNPs against *Pseudomonas florescence, Proteus spp.* and *Flavobacterium spp.* of marine aquatic pathogen were confirmed by the circular inhibition zone formed around the well impregnated with different concentration of AgNPs. The antimicrobial effect varies according to the species and the effect was higher at 75 μg/μL concentration of AgNPs. A maximum zone was recorded as 16, 14 and 14 mm for *Pseudomonas florescence, Proteus spp.*, and *Flavobacterium spp.* respectively at 75 μg/μL concentrations. A maximum zone was also recorded as 17, 14 and 15 mm for *Pseudomonas florescence, Proteus spp.*, and *Flavobacterium spp.* respectively when treated with Chloramphenicol (1 ppm) as a control. No zone formations were observed for the plant extract alone. Thus the zone observed in control was significantly equal at 75 μg/μL concentration that confirms the antimicrobial effect of biosynthesized AgNPs to control the marine microbial pathogens and the Figure 5 shows the rate of inhibition against marine ornamental fish pathogens. The antibacterial effect of AgNPs inside bacterial cells will form a strong association with bacterial cellular components. Once inside the cell, nanoparticles would interfere with the bacterial growth signaling pathway by modulating tyrosine phosphorylation of putative peptide substrates critical for cell viability and division. Inside a bacterium, nanoparticles can interact with DNA, thus losing its ability to replicate which may lead to the cell death. Interaction between such nanoparticles and the cell wall of bacteria would be facilitated by the relative abundance of negative charges on the gram-negative bacteria, which was affable to the fact that growth of gram-negative bacteria was more profoundly affected by the AgNPs than that of the gram-positive organisms [32,33] Thus Figure 5 represents the maximum zone formation was found in *Pseudomonas fluorescence* highest zone formation in 16 mm at 75 μg/μL, 14 mm at 50 μg/μL and 12 mm in 25 μg/μL.

## Conclusion

It is concluded that the present study reveals the simple, efficient and eco-friendly one pot green synthesis of AgNPs using mangrove leaf buds extract. Though there was report by Gnanadesigan et al. 2011 [34], in this work the AgNPs were prepared in one pot green synthesis within 5 minutes which was spherical in shape with an average size of 4 nm and stable at room temperature for more than a year. The present study also reports the antimicrobial activity of AgNPs against marine ornamental fish pathogens such as *Proteus spp.Pseudomonas florescence* and *Flavobacterium spp.,* isolated from an infected fish, *Dascyllus trimaculatus*. The use of antibiotics in marine ornamental fishes can lead to the development of antibiotic-resistant bacterial strains. Thus this report confirmed a promising alternative approach for controlling marine ornamental fish diseases by the use of AgNPs in place of synthetic antibiotics. Thus the biosynthesized AgNPs can also be included among the potential biological disease controlling agent in aquatic pathogens.

## Competing interests

The authors declare that they have no competing interests.

## Authors’ contributions

One pot green synthesis was done by JU. Nanoparticles characterization (UV, TEM, XRD, FTIR) and results interpretation was done by DI. Pathogen isolation and Antibacterial assay was done by JU, TTA and TB. Manuscript preparation was done by JU & DI. As a whole the entire work was carried out by JU under the guidance of DI. Permission for research was given by TTA & TB. All authors read and approved the final manuscript.
